# Tris(1,10-phenanthroline-κ^2^ *N*,*N*′)zinc(II) chloride 2-phenyl-4-selenazole-5-car­box­yl­ate decahydrate

**DOI:** 10.1107/S1600536811000729

**Published:** 2011-01-15

**Authors:** Jin-Bei Shen, Xin Lv, Ji-Fei Chen, Yu-Feng Zhou, Guo-Liang Zhao

**Affiliations:** a College of Chemistry and Life Science, Zhejiang Normal University, Jinhua 321004, Zhejiang, People’s Republic of China; b Zhejiang Normal University Xingzhi College, Jinhua, Zhejiang 321004, People’s Republic of China

## Abstract

The asymmetric unit of the title salt, [Zn(C_12_H_8_N_2_)_3_](C_10_H_6_NO_2_Se)Cl·10H_2_O, contains a [Zn(phen)_3_]^2+^ cation (phen is 1,10-phenanthroline), uncoordinated chloride and 2-phenyl-4-selenazole-5-carboxyl­ate anions and ten uncoord­in­ated water mol­ecules. The central Zn^II^ ion is six-coordinated by six N atoms from three phen ligands in a distorted octa­hedral geometry. An extensive O—H⋯O, O—H⋯N and O—H⋯Cl hydrogen-bonding network stabilizes the crystal structure.

## Related literature

For the synthesis of the organic ligand, 2-phenyl-4-selenazole-5-carboxylic acid, see: Zhao *et al.* (2010 ▶). For related structures, see: Srivastava & Robins (1983 ▶); Boritzki *et al.* (1985 ▶); Wang *et al.* (2006 ▶).
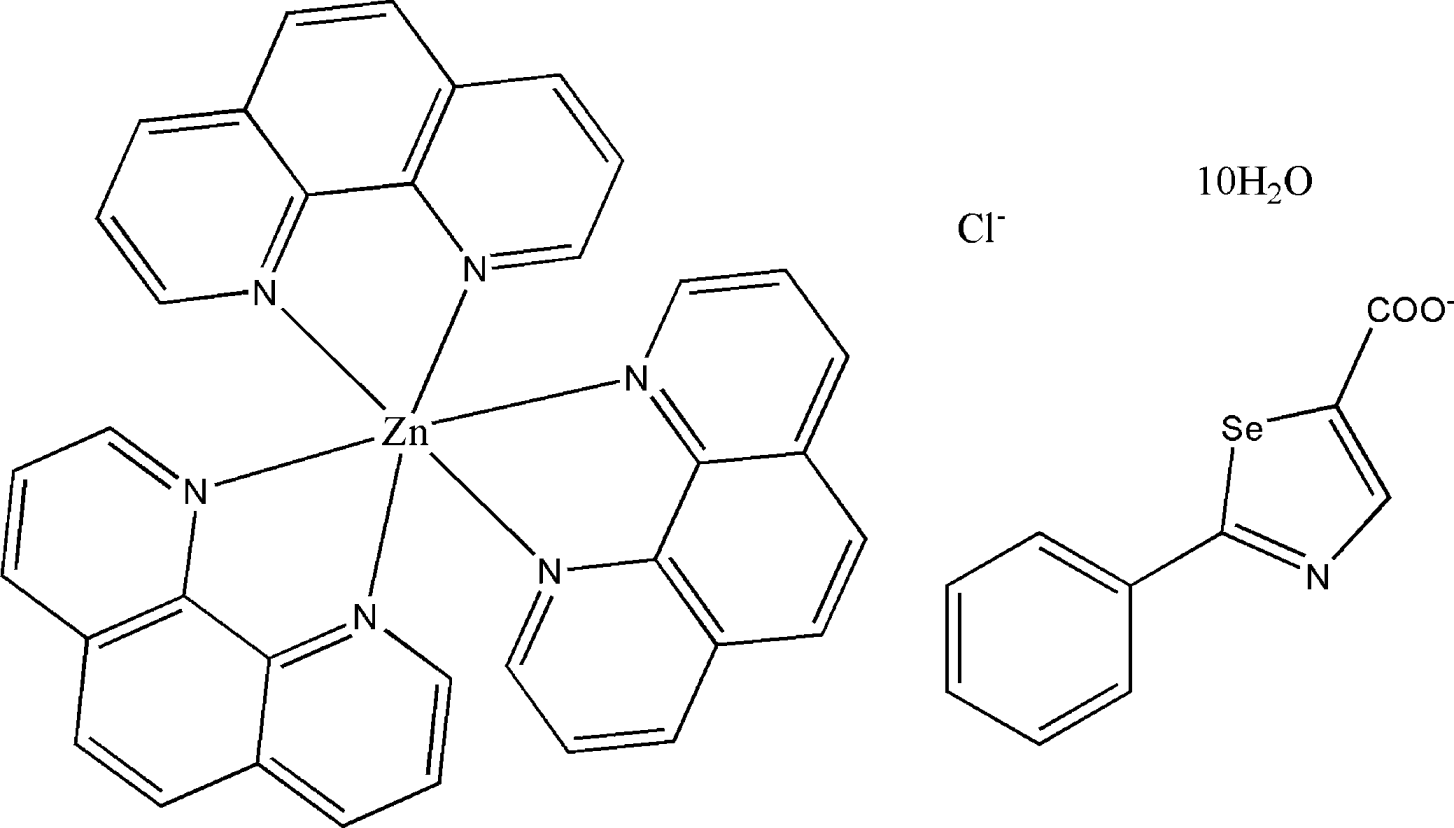


## Experimental

### 

#### Crystal data

[Zn(C_12_H_8_N_2_)_3_](C_10_H_6_NO_2_Se)Cl·10H_2_O
*M* *_r_* = 1072.71
Triclinic, 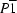

*a* = 12.4837 (9) Å
*b* = 13.8935 (10) Å
*c* = 15.8221 (12) Å
α = 77.618 (4)°
β = 89.642 (4)°
γ = 63.375 (4)°
*V* = 2383.4 (3) Å^3^
*Z* = 2
Mo *K*α radiation
μ = 1.40 mm^−1^
*T* = 296 K
0.64 × 0.35 × 0.17 mm

#### Data collection

Bruker APEXII area-detector diffractometer
Absorption correction: multi-scan (*SADABS*; Sheldrick, 1996 ▶) *T*_min_ = 0.562, *T*_max_ = 0.790
33787 measured reflections
8371 independent reflections
6305 reflections with *I* > 2σ(*I*)
*R*_int_ = 0.038

#### Refinement

*R*[*F* ^2^ > 2σ(*F* ^2^)] = 0.047
*wR*(*F* ^2^) = 0.140
*S* = 1.06
8371 reflections
613 parameters
30 restraints
H-atom parameters constrained
Δρ_max_ = 0.81 e Å^−3^
Δρ_min_ = −0.74 e Å^−3^

### 

Data collection: *APEX2* (Bruker, 2006 ▶); cell refinement: *SAINT* (Bruker, 2006 ▶); data reduction: *SAINT*; program(s) used to solve structure: *SHELXS97* (Sheldrick, 2008 ▶); program(s) used to refine structure: *SHELXL97* (Sheldrick, 2008 ▶); molecular graphics: *SHELXTL* (Sheldrick, 2008 ▶); software used to prepare material for publication: *SHELXL97*.

## Supplementary Material

Crystal structure: contains datablocks I, global. DOI: 10.1107/S1600536811000729/hg2778sup1.cif

Structure factors: contains datablocks I. DOI: 10.1107/S1600536811000729/hg2778Isup2.hkl

Additional supplementary materials:  crystallographic information; 3D view; checkCIF report

## Figures and Tables

**Table 1:** Hydrogen-bond geometry (Å, °)

*D*—H⋯*A*	*D*—H	H⋯*A*	*D*⋯*A*	*D*—H⋯*A*
O1*W*—H1*WA*⋯O2*W*^i^	0.85	2.05	2.898 (5)	179
O1*W*—H1*WB*⋯O9*W*^ii^	0.85	1.98	2.830 (6)	180
O4*W*—H4*WA*⋯N7^ii^	0.85	2.22	3.044 (5)	163
O4*W*—H4*WA*⋯O1^ii^	0.85	2.40	2.921 (7)	120
O5*W*—H5*WA*⋯O6*W*^iii^	0.85	1.91	2.762 (6)	178
O6*W*—H6*WA*⋯Cl1^iii^	0.85	2.24	3.076 (5)	168
O7*W*—H7*WB*⋯Cl1^iv^	0.85	2.54	3.391 (5)	179
O8*W*—H8*WB*⋯O7*W*^v^	0.85	1.97	2.817 (7)	180
O2*W*—H2*WA*⋯O9*W*^vi^	0.85	1.96	2.806 (5)	179
O2*W*—H2*WB*⋯Cl1^vii^	0.85	2.28	3.129 (3)	180
O4*W*—H4*WB*⋯O1	0.85	1.98	2.809 (6)	166
O5*W*—H5*WB*⋯O3*W*	0.85	2.07	2.915 (6)	173
O6*W*—H6*WB*⋯O2	0.85	2.05	2.892 (6)	171
O7*W*—H7*WA*⋯Cl1	0.85	2.24	3.006 (5)	150
O8*W*—H8*WA*⋯O2	0.85	1.97	2.819 (5)	172
O9*W*—H9*WA*⋯O8*W*	0.85	2.05	2.864 (8)	161
O9*W*—H9*WB*⋯O10*W*	0.85	2.18	3.030 (8)	178
O10*W*—H10*W*⋯O4*W*	0.85	2.02	2.866 (8)	173
O10*W*—H10*E*⋯O2	0.85	2.08	2.925 (6)	171

Symmetry codes: (i) 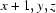; (ii) 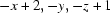; (iii) 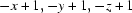; (iv) 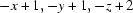; (v) 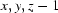; (vi) 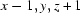; (vii) 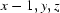.

**Fig. 1.**
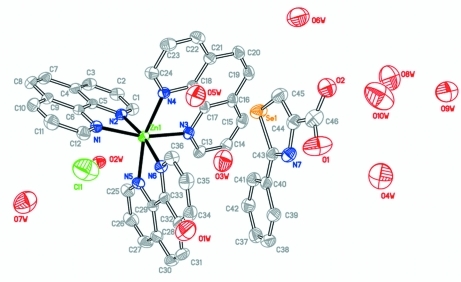
The molecular structure of the title complex, showing the atom- labeling scheme. Displacement ellipsoids are drawn at the 30% probability level.

**Fig. 2.**
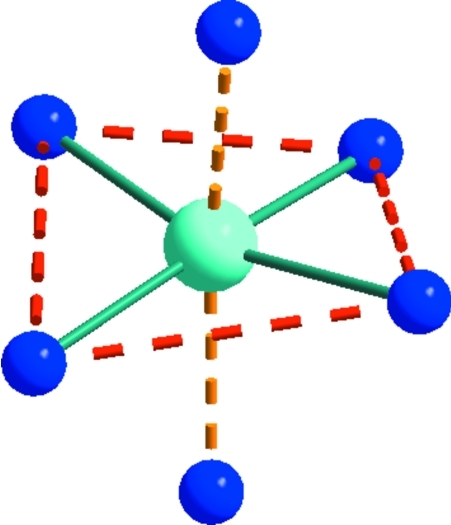
The coordination environment of the ZnII atom, showing the octahedral structurem.

**Fig. 3.**
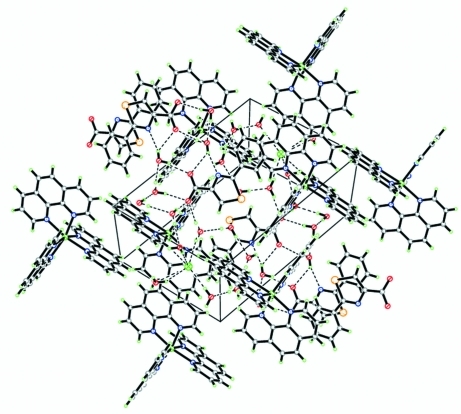
The stacking plot of the title compound, showing H-bond interactions (dashed lines) and π···π stacking interactions.

## supplementary crystallographic information

### Comment

Derivatives of selenazole are important in chemistry and biochemistry due to their biological activity (Srivastava *et al.*, 1983; Boritzki *et al.*, 1985). Interested in this field, we have been engaged in a major effort directed toward the development of syntheses of new selenazole carboxylic acid and their transition metal complexes. We have reported our partial research results previously (Zhao *et al.*, 2010). Herein, we describe the structure of the title Zn^II^ complex.

The local coordination geometry around each Zn center (Fig. 1 and Fig. 2) is a slightly distorted octahedron defined by six nitrogen atoms from three different 1,10-phenanthroline (phen) ligands while the organic ligand 2-phenyl-4- selenazole carboxylic acid (H*L*) loses its one proton and changes to *L* which fail to coordinate to zinc atom.That is, the compound consists of [Zn(phen)_3_]^2+^ cations, two un-coordinating anions: Cl^-^and *L* and ten water molecules.  The bonds between Zn^II^ and N atoms from 1, 10-phenanthroline are 2.155 (3)Å - 2.185 (3)Å, which are similar to related compounds in the literatures (Wang *et al.* 2006).  The three phen ligands around zinc is respectively plane,the four N atoms of phen form the equatorial plane, and N4 and N5 atoms occupy the apical positions.

The hydrogen bonds and π···π weak non-covalent interactions lend stability to the structure. The hydrogen bonds are listed in Table 2 and the stacking plot of this compound is shown in Fig. 3. Complex molecules are linked in a line through water molecules by hydrogen bonds and different lines are interlocked with benzene rings using π···π stacking.

### Experimental

Reagents and solvents used were of commercially available quality and without purification before using. The compound (1) was obtained by adding ZnCl_2_ (0.136 g,1 mmol) to 2-phenyl-4-selenazole carboxylic acid (0.252 g,1 mmol),1,10- phenanthroline (0.396 g, 2 mmol) in ethanol solution. The mixture was stirred at room temperature for 8 h to obtain a colourless solution which was filtered and the filtrate kept for evaporating. colourless crystal of the title complex formed after 40 days.

### Refinement

The H atoms bonded to C atoms were positioned geometrically and refined using a riding model [aromatic C—H = 0.93 Å (*U*_iso_(H) = 1.2*U*_eq_(C))]. Water H atoms bonded to O atoms were located in difference Fourier maps and refined with O—H distance restraints of 0.83 (2) and *U*_iso_(H) = 1.5*U*_eq_(O).

### Crystal data

**Table:**

[Zn(C_12_H_8_N_2_)_3_](C_10_H_6_NO_2_Se)Cl·10H_2_O	*Z* = 2
*M**_r_* = 1072.71	*F*(000) = 1104
Triclinic, *P*1	*D*_x_ = 1.495 Mg m^−^^3^
Hall symbol: -P 1	Mo *K*α radiation, λ = 0.71073 Å
*a* = 12.4837 (9) Å	Cell parameters from 8933 reflections
*b* = 13.8935 (10) Å	θ = 1.8–25.0°
*c* = 15.8221 (12) Å	µ = 1.40 mm^−^^1^
α = 77.618 (4)°	*T* = 296 K
β = 89.642 (4)°	Block, colourless
γ = 63.375 (4)°	0.64 × 0.35 × 0.17 mm
*V* = 2383.4 (3) Å^3^	

### Data collection

**Table:**

Bruker APEXII area-detector diffractometer	8371 independent reflections
Radiation source: fine-focus sealed tube	6305 reflections with *I* > 2σ(*I*)
graphite	*R*_int_ = 0.038
φ and ω scans	θ_max_ = 25.0°, θ_min_ = 1.8°
Absorption correction: multi-scan (*SADABS*; Sheldrick, 1996)	*h* = −14→14
*T*_min_ = 0.562, *T*_max_ = 0.790	*k* = −16→16
33787 measured reflections	*l* = −18→18

### Refinement

**Table:**

Refinement on *F*^2^	Primary atom site location: structure-invariant direct methods
Least-squares matrix: full	Secondary atom site location: difference Fourier map
*R*[*F*^2^ > 2σ(*F*^2^)] = 0.047	Hydrogen site location: inferred from neighbouring sites
*wR*(*F*^2^) = 0.140	H-atom parameters constrained
*S* = 1.06	*w* = 1/[σ^2^(*F*_o_^2^) + (0.0806*P*)^2^ + 0.8651*P*] where *P* = (*F*_o_^2^ + 2*F*_c_^2^)/3
8371 reflections	(Δ/σ)_max_ < 0.001
613 parameters	Δρ_max_ = 0.81 e Å^−^^3^
30 restraints	Δρ_min_ = −0.74 e Å^−^^3^

### Special details

**Table:**

Geometry. All e.s.d.'s (except the e.s.d. in the dihedral angle between two l.s. planes) are estimated using the full covariance matrix. The cell e.s.d.'s are taken into account individually in the estimation of e.s.d.'s in distances, angles and torsion angles; correlations between e.s.d.'s in cell parameters are only used when they are defined by crystal symmetry. An approximate (isotropic) treatment of cell e.s.d.'s is used for estimating e.s.d.'s involving l.s. planes.
Refinement. Refinement of *F*^2^ against ALL reflections. The weighted *R*-factor *wR* and goodness of fit *S* are based on *F*^2^, conventional *R*-factors *R* are based on *F*, with *F* set to zero for negative *F*^2^. The threshold expression of *F*^2^ > σ(*F*^2^) is used only for calculating *R*-factors(gt) *etc*. and is not relevant to the choice of reflections for refinement. *R*-factors based on *F*^2^ are statistically about twice as large as those based on *F*, and *R*- factors based on ALL data will be even larger.

### Fractional atomic coordinates and isotropic or equivalent isotropic displacement parameters (Å^2^)

**Table:**

	*x*	*y*	*z*	*U*_iso_*/*U*_eq_	
Zn1	0.19692 (3)	0.30045 (3)	0.77746 (2)	0.03857 (14)	
Se1	0.43865 (4)	0.23586 (4)	0.49471 (3)	0.07071 (17)	
N1	0.1832 (3)	0.4178 (2)	0.85189 (18)	0.0424 (7)	
N3	0.1650 (2)	0.2219 (2)	0.68130 (18)	0.0415 (7)	
N4	0.1851 (2)	0.4125 (2)	0.65436 (17)	0.0390 (6)	
C43	0.5785 (3)	0.1029 (3)	0.5404 (2)	0.0471 (9)	
N2	0.0048 (3)	0.3822 (2)	0.79275 (18)	0.0412 (7)	
C18	0.1620 (3)	0.3806 (3)	0.5840 (2)	0.0385 (8)	
N5	0.2362 (3)	0.1535 (3)	0.87845 (18)	0.0446 (7)	
N6	0.3907 (3)	0.2104 (2)	0.78710 (18)	0.0428 (7)	
C29	0.3519 (3)	0.0743 (3)	0.8845 (2)	0.0429 (8)	
C17	0.1493 (3)	0.2808 (3)	0.5982 (2)	0.0405 (8)	
C5	−0.0267 (3)	0.4672 (3)	0.8330 (2)	0.0398 (8)	
C33	0.4351 (3)	0.1060 (3)	0.8375 (2)	0.0411 (8)	
C4	−0.1461 (3)	0.5338 (3)	0.8457 (2)	0.0484 (9)	
N7	0.6756 (3)	0.1018 (3)	0.5097 (2)	0.0532 (8)	
C21	0.1516 (3)	0.4420 (3)	0.4983 (2)	0.0470 (9)	
C16	0.1221 (3)	0.2475 (3)	0.5270 (2)	0.0490 (9)	
C40	0.5741 (3)	0.0113 (3)	0.6027 (2)	0.0479 (9)	
C36	0.4676 (3)	0.2405 (4)	0.7452 (3)	0.0534 (10)	
H36A	0.4381	0.3118	0.7101	0.064*	
C6	0.0684 (3)	0.4867 (3)	0.8636 (2)	0.0420 (8)	
C25	0.1598 (4)	0.1265 (4)	0.9235 (2)	0.0565 (10)	
H25A	0.0806	0.1805	0.9209	0.068*	
C13	0.1554 (3)	0.1289 (3)	0.6952 (3)	0.0480 (9)	
H13A	0.1675	0.0875	0.7520	0.058*	
C24	0.2010 (3)	0.5024 (3)	0.6411 (2)	0.0505 (9)	
H24A	0.2170	0.5243	0.6891	0.061*	
C32	0.5577 (3)	0.0283 (3)	0.8460 (2)	0.0505 (9)	
C1	−0.0815 (3)	0.3633 (3)	0.7646 (2)	0.0479 (9)	
H1A	−0.0605	0.3052	0.7373	0.057*	
C11	0.2502 (4)	0.5211 (4)	0.9210 (3)	0.0649 (12)	
H11A	0.3144	0.5312	0.9392	0.078*	
C28	0.3934 (4)	−0.0346 (3)	0.9353 (2)	0.0526 (9)	
C12	0.2705 (4)	0.4352 (3)	0.8807 (2)	0.0531 (9)	
H12A	0.3495	0.3879	0.8738	0.064*	
C14	0.1281 (4)	0.0904 (4)	0.6283 (3)	0.0623 (11)	
H14A	0.1219	0.0247	0.6408	0.075*	
C3	−0.2354 (3)	0.5099 (4)	0.8149 (3)	0.0576 (10)	
H3A	−0.3157	0.5515	0.8227	0.069*	
C41	0.4668 (4)	0.0119 (4)	0.6252 (3)	0.0572 (10)	
H41A	0.3944	0.0727	0.6000	0.069*	
C31	0.5950 (4)	−0.0802 (4)	0.8985 (3)	0.0669 (12)	
H31A	0.6759	−0.1316	0.9042	0.080*	
O2	0.7391 (3)	0.2748 (3)	0.3355 (2)	0.0776 (9)	
C9	0.0390 (4)	0.5745 (3)	0.9049 (2)	0.0515 (9)	
C44	0.6545 (4)	0.1973 (3)	0.4470 (3)	0.0531 (10)	
C34	0.6337 (4)	0.0650 (4)	0.8007 (3)	0.0630 (12)	
H34A	0.7154	0.0167	0.8045	0.076*	
C20	0.1236 (3)	0.4051 (4)	0.4277 (2)	0.0562 (10)	
H20A	0.1158	0.4455	0.3711	0.067*	
C2	−0.2036 (3)	0.4265 (4)	0.7740 (3)	0.0564 (10)	
H2A	−0.2619	0.4111	0.7524	0.068*	
C26	0.1939 (5)	0.0196 (4)	0.9748 (3)	0.0694 (13)	
H26A	0.1377	0.0035	1.0052	0.083*	
C45	0.5393 (4)	0.2780 (4)	0.4290 (3)	0.0664 (11)	
H45A	0.5144	0.3456	0.3890	0.080*	
C46	0.7594 (4)	0.2044 (4)	0.4063 (4)	0.0693 (12)	
C30	0.5170 (5)	−0.1111 (4)	0.9403 (3)	0.0679 (12)	
H30A	0.5444	−0.1834	0.9729	0.081*	
C22	0.1704 (3)	0.5354 (4)	0.4878 (3)	0.0565 (10)	
H22A	0.1663	0.5770	0.4323	0.068*	
C35	0.5906 (4)	0.1693 (4)	0.7516 (3)	0.0631 (11)	
H35A	0.6423	0.1935	0.7225	0.076*	
C39	0.6816 (4)	−0.0817 (4)	0.6421 (3)	0.0593 (11)	
H39A	0.7553	−0.0843	0.6285	0.071*	
C23	0.1947 (4)	0.5654 (4)	0.5584 (3)	0.0599 (10)	
H23A	0.2071	0.6277	0.5518	0.072*	
C42	0.4650 (4)	−0.0758 (4)	0.6844 (3)	0.0633 (11)	
H42A	0.3917	−0.0734	0.6991	0.076*	
C15	0.1106 (4)	0.1492 (4)	0.5446 (3)	0.0612 (11)	
H15A	0.0911	0.1246	0.4995	0.073*	
C7	−0.1715 (4)	0.6205 (3)	0.8894 (3)	0.0625 (11)	
H7A	−0.2506	0.6642	0.8987	0.075*	
C19	0.1083 (3)	0.3126 (4)	0.4414 (3)	0.0573 (11)	
H19A	0.0886	0.2915	0.3942	0.069*	
C10	0.1353 (5)	0.5901 (4)	0.9334 (3)	0.0633 (11)	
H10A	0.1206	0.6473	0.9606	0.076*	
C27	0.3092 (5)	−0.0600 (4)	0.9797 (3)	0.0677 (12)	
H27A	0.3320	−0.1314	1.0127	0.081*	
C8	−0.0838 (4)	0.6399 (3)	0.9172 (3)	0.0651 (12)	
H8A	−0.1032	0.6973	0.9451	0.078*	
O1	0.8605 (3)	0.1404 (4)	0.4456 (3)	0.1188 (16)	
C37	0.5696 (5)	−0.1658 (4)	0.7214 (3)	0.0678 (12)	
H37A	0.5681	−0.2255	0.7605	0.081*	
C38	0.6784 (4)	−0.1683 (4)	0.7005 (3)	0.0672 (12)	
H38A	0.7501	−0.2295	0.7265	0.081*	
Cl1	0.57973 (16)	0.37700 (19)	0.91079 (13)	0.1335 (7)	
O3W	0.8109 (3)	0.2373 (3)	0.6383 (2)	0.0912 (11)	
H3WA	0.8472	0.2030	0.6890	0.137*	
H3WB	0.8205	0.2095	0.5942	0.137*	
O1W	0.9146 (4)	0.1104 (3)	0.8120 (3)	0.1032 (12)	
H1WA	0.8967	0.1422	0.8541	0.155*	
H1WB	0.9591	0.0409	0.8288	0.155*	
O4W	1.0793 (4)	0.0140 (4)	0.3841 (3)	0.1190 (15)	
H4WA	1.1390	−0.0158	0.4228	0.178*	
H4WB	1.0199	0.0472	0.4113	0.178*	
O5W	0.5718 (4)	0.4248 (4)	0.5881 (3)	0.1123 (13)	
H5WA	0.5347	0.4502	0.6298	0.168*	
H5WB	0.6384	0.3665	0.6041	0.168*	
O6W	0.5449 (4)	0.4965 (4)	0.2741 (3)	0.1146 (14)	
H6WA	0.5182	0.5230	0.2206	0.172*	
H6WB	0.6067	0.4337	0.2885	0.172*	
O7W	0.4968 (4)	0.3559 (4)	1.0895 (3)	0.1256 (16)	
H7WA	0.5454	0.3512	1.0509	0.188*	
H7WB	0.4776	0.4228	1.0899	0.188*	
O8W	0.7050 (5)	0.1907 (4)	0.1966 (3)	0.1297 (16)	
H8WA	0.7207	0.2171	0.2358	0.195*	
H8WB	0.6421	0.2405	0.1642	0.195*	
O9W	0.9370 (6)	0.1210 (4)	0.1330 (3)	0.160 (2)	
H9WA	0.8672	0.1309	0.1461	0.240*	
H9WB	0.9456	0.1509	0.1723	0.240*	
O10W	0.9719 (5)	0.2306 (6)	0.2694 (3)	0.155 (2)	
H10W	1.0099	0.1656	0.3016	0.233*	
H10E	0.9087	0.2362	0.2930	0.233*	
O2W	−0.1445 (3)	0.2174 (2)	0.95610 (18)	0.0604 (7)	
H2WA	−0.1192	0.1879	1.0096	0.091*	
H2WB	−0.2194	0.2607	0.9438	0.091*	

### Atomic displacement parameters (Å^2^)

**Table:**

	*U*^11^	*U*^22^	*U*^33^	*U*^12^	*U*^13^	*U*^23^
Zn1	0.0347 (2)	0.0370 (2)	0.0420 (2)	−0.01497 (18)	0.00388 (16)	−0.00878 (17)
Se1	0.0395 (2)	0.0629 (3)	0.0806 (3)	−0.0045 (2)	0.0095 (2)	−0.0029 (2)
N1	0.0410 (16)	0.0416 (17)	0.0411 (15)	−0.0164 (14)	0.0033 (13)	−0.0085 (13)
N3	0.0361 (15)	0.0433 (18)	0.0481 (17)	−0.0183 (14)	0.0080 (13)	−0.0165 (14)
N4	0.0354 (15)	0.0371 (16)	0.0425 (15)	−0.0157 (13)	0.0010 (12)	−0.0074 (13)
C43	0.0333 (18)	0.049 (2)	0.052 (2)	−0.0101 (17)	0.0014 (16)	−0.0186 (18)
N2	0.0404 (16)	0.0392 (17)	0.0434 (16)	−0.0186 (14)	0.0043 (13)	−0.0074 (13)
C18	0.0265 (16)	0.041 (2)	0.0422 (18)	−0.0105 (15)	0.0058 (13)	−0.0100 (15)
N5	0.0433 (17)	0.0469 (18)	0.0429 (16)	−0.0221 (15)	0.0024 (13)	−0.0058 (13)
N6	0.0393 (16)	0.0419 (18)	0.0472 (16)	−0.0190 (14)	0.0048 (13)	−0.0098 (14)
C29	0.050 (2)	0.042 (2)	0.0384 (18)	−0.0218 (18)	−0.0036 (15)	−0.0111 (16)
C17	0.0262 (16)	0.046 (2)	0.046 (2)	−0.0111 (15)	0.0071 (14)	−0.0174 (16)
C5	0.0414 (19)	0.0333 (19)	0.0374 (17)	−0.0126 (16)	0.0058 (15)	−0.0039 (15)
C33	0.0406 (19)	0.039 (2)	0.0416 (18)	−0.0138 (16)	−0.0036 (15)	−0.0146 (16)
C4	0.047 (2)	0.037 (2)	0.046 (2)	−0.0092 (17)	0.0084 (16)	−0.0028 (16)
N7	0.0403 (17)	0.050 (2)	0.068 (2)	−0.0178 (15)	0.0035 (15)	−0.0182 (17)
C21	0.0305 (18)	0.051 (2)	0.045 (2)	−0.0086 (16)	0.0049 (15)	−0.0067 (17)
C16	0.0325 (18)	0.059 (3)	0.055 (2)	−0.0146 (18)	0.0052 (16)	−0.0284 (19)
C40	0.040 (2)	0.051 (2)	0.048 (2)	−0.0142 (18)	0.0057 (16)	−0.0190 (18)
C36	0.046 (2)	0.056 (3)	0.061 (2)	−0.026 (2)	0.0084 (18)	−0.0108 (19)
C6	0.053 (2)	0.0327 (19)	0.0343 (17)	−0.0168 (17)	0.0033 (15)	−0.0026 (14)
C25	0.062 (3)	0.063 (3)	0.054 (2)	−0.038 (2)	0.0082 (19)	−0.008 (2)
C13	0.044 (2)	0.041 (2)	0.063 (2)	−0.0216 (18)	0.0098 (17)	−0.0150 (18)
C24	0.054 (2)	0.048 (2)	0.051 (2)	−0.027 (2)	−0.0037 (17)	−0.0061 (18)
C32	0.044 (2)	0.046 (2)	0.053 (2)	−0.0093 (18)	−0.0050 (17)	−0.0198 (18)
C1	0.041 (2)	0.049 (2)	0.055 (2)	−0.0230 (18)	0.0069 (17)	−0.0091 (17)
C11	0.079 (3)	0.067 (3)	0.062 (3)	−0.046 (3)	−0.006 (2)	−0.012 (2)
C28	0.069 (3)	0.041 (2)	0.047 (2)	−0.026 (2)	−0.0056 (19)	−0.0085 (17)
C12	0.055 (2)	0.057 (3)	0.052 (2)	−0.030 (2)	−0.0011 (18)	−0.0116 (19)
C14	0.058 (3)	0.058 (3)	0.088 (3)	−0.035 (2)	0.017 (2)	−0.034 (2)
C3	0.037 (2)	0.052 (3)	0.071 (3)	−0.0132 (18)	0.0131 (18)	−0.005 (2)
C41	0.041 (2)	0.061 (3)	0.065 (3)	−0.017 (2)	0.0060 (18)	−0.019 (2)
C31	0.062 (3)	0.041 (2)	0.075 (3)	−0.002 (2)	−0.017 (2)	−0.017 (2)
O2	0.075 (2)	0.083 (2)	0.080 (2)	−0.0430 (19)	0.0178 (18)	−0.0158 (19)
C9	0.069 (3)	0.033 (2)	0.046 (2)	−0.0183 (19)	0.0025 (18)	−0.0083 (16)
C44	0.049 (2)	0.053 (3)	0.061 (2)	−0.024 (2)	0.0054 (18)	−0.020 (2)
C34	0.036 (2)	0.071 (3)	0.074 (3)	−0.011 (2)	0.005 (2)	−0.032 (3)
C20	0.045 (2)	0.066 (3)	0.043 (2)	−0.012 (2)	0.0055 (17)	−0.0138 (19)
C2	0.040 (2)	0.059 (3)	0.069 (3)	−0.026 (2)	0.0050 (18)	−0.006 (2)
C26	0.090 (4)	0.074 (3)	0.060 (3)	−0.057 (3)	0.007 (2)	−0.003 (2)
C45	0.057 (3)	0.055 (3)	0.076 (3)	−0.018 (2)	0.008 (2)	−0.011 (2)
C46	0.060 (3)	0.063 (3)	0.095 (4)	−0.035 (3)	0.009 (3)	−0.023 (3)
C30	0.090 (3)	0.037 (2)	0.063 (3)	−0.018 (2)	−0.016 (2)	−0.008 (2)
C22	0.048 (2)	0.061 (3)	0.049 (2)	−0.022 (2)	0.0046 (18)	0.0008 (19)
C35	0.043 (2)	0.078 (3)	0.073 (3)	−0.028 (2)	0.017 (2)	−0.026 (3)
C39	0.041 (2)	0.064 (3)	0.058 (2)	−0.012 (2)	0.0080 (18)	−0.013 (2)
C23	0.059 (3)	0.049 (2)	0.070 (3)	−0.029 (2)	0.004 (2)	−0.003 (2)
C42	0.062 (3)	0.065 (3)	0.065 (3)	−0.029 (2)	0.011 (2)	−0.018 (2)
C15	0.053 (2)	0.068 (3)	0.073 (3)	−0.027 (2)	0.006 (2)	−0.036 (2)
C7	0.057 (3)	0.045 (2)	0.062 (3)	−0.004 (2)	0.015 (2)	−0.009 (2)
C19	0.042 (2)	0.075 (3)	0.048 (2)	−0.014 (2)	0.0008 (17)	−0.028 (2)
C10	0.089 (3)	0.044 (2)	0.058 (2)	−0.030 (2)	−0.002 (2)	−0.015 (2)
C27	0.097 (4)	0.051 (3)	0.056 (2)	−0.043 (3)	−0.013 (2)	0.005 (2)
C8	0.078 (3)	0.038 (2)	0.060 (3)	−0.008 (2)	0.008 (2)	−0.0154 (19)
O1	0.049 (2)	0.100 (3)	0.180 (4)	−0.034 (2)	0.011 (2)	0.020 (3)
C37	0.084 (3)	0.063 (3)	0.058 (3)	−0.033 (3)	0.019 (2)	−0.017 (2)
C38	0.057 (3)	0.057 (3)	0.063 (3)	−0.009 (2)	0.005 (2)	−0.005 (2)
Cl1	0.0872 (11)	0.1591 (19)	0.1237 (14)	−0.0427 (12)	0.0137 (10)	−0.0052 (12)
O3W	0.096 (3)	0.092 (3)	0.098 (3)	−0.048 (2)	0.028 (2)	−0.035 (2)
O1W	0.107 (3)	0.078 (3)	0.112 (3)	−0.030 (2)	0.015 (2)	−0.025 (2)
O4W	0.082 (3)	0.157 (4)	0.128 (3)	−0.056 (3)	−0.011 (2)	−0.051 (3)
O5W	0.109 (3)	0.104 (3)	0.110 (3)	−0.034 (3)	0.023 (3)	−0.033 (3)
O6W	0.107 (3)	0.090 (3)	0.119 (3)	−0.029 (3)	0.023 (3)	−0.008 (2)
O7W	0.088 (3)	0.120 (4)	0.152 (4)	−0.031 (3)	0.036 (3)	−0.037 (3)
O8W	0.145 (4)	0.154 (5)	0.108 (3)	−0.076 (4)	0.011 (3)	−0.047 (3)
O9W	0.207 (6)	0.100 (4)	0.105 (3)	−0.013 (4)	−0.001 (4)	−0.020 (3)
O10W	0.115 (4)	0.222 (6)	0.128 (4)	−0.096 (4)	0.022 (3)	0.003 (4)
O2W	0.0598 (17)	0.0520 (17)	0.0704 (18)	−0.0283 (14)	0.0021 (14)	−0.0102 (14)

### Geometric parameters (Å, °)

**Table:**

Zn1—N1	2.155 (3)	C3—C2	1.351 (6)
Zn1—N6	2.156 (3)	C3—H3A	0.9300
Zn1—N5	2.163 (3)	C41—C42	1.378 (6)
Zn1—N4	2.176 (3)	C41—H41A	0.9300
Zn1—N2	2.184 (3)	C31—C30	1.348 (7)
Zn1—N3	2.185 (3)	C31—H31A	0.9300
Se1—C45	1.840 (5)	O2—C46	1.260 (6)
Se1—C43	1.880 (4)	C9—C10	1.405 (6)
N1—C12	1.320 (5)	C9—C8	1.428 (6)
N1—C6	1.360 (5)	C44—C45	1.353 (6)
N3—C13	1.323 (4)	C44—C46	1.488 (6)
N3—C17	1.355 (4)	C34—C35	1.348 (6)
N4—C24	1.323 (5)	C34—H34A	0.9300
N4—C18	1.360 (4)	C20—C19	1.354 (6)
C43—N7	1.298 (5)	C20—H20A	0.9300
C43—C40	1.456 (6)	C2—H2A	0.9300
N2—C1	1.320 (4)	C26—C27	1.356 (7)
N2—C5	1.363 (4)	C26—H26A	0.9300
C18—C21	1.410 (5)	C45—H45A	0.9300
C18—C17	1.435 (5)	C46—O1	1.240 (6)
N5—C25	1.324 (5)	C30—H30A	0.9300
N5—C29	1.356 (5)	C22—C23	1.354 (6)
N6—C36	1.330 (5)	C22—H22A	0.9300
N6—C33	1.355 (5)	C35—H35A	0.9300
C29—C28	1.408 (5)	C39—C38	1.368 (6)
C29—C33	1.444 (5)	C39—H39A	0.9300
C17—C16	1.402 (5)	C23—H23A	0.9300
C5—C4	1.399 (5)	C42—C37	1.358 (6)
C5—C6	1.439 (5)	C42—H42A	0.9300
C33—C32	1.408 (5)	C15—H15A	0.9300
C4—C3	1.414 (6)	C7—C8	1.337 (6)
C4—C7	1.429 (6)	C7—H7A	0.9300
N7—C44	1.393 (5)	C19—H19A	0.9300
C21—C22	1.396 (6)	C10—H10A	0.9300
C21—C20	1.430 (5)	C27—H27A	0.9300
C16—C15	1.407 (6)	C8—H8A	0.9300
C16—C19	1.422 (6)	C37—C38	1.382 (6)
C40—C41	1.380 (5)	C37—H37A	0.9300
C40—C39	1.403 (6)	C38—H38A	0.9300
C36—C35	1.395 (6)	O3W—H3WA	0.8496
C36—H36A	0.9300	O3W—H3WB	0.8502
C6—C9	1.410 (5)	O1W—H1WA	0.8502
C25—C26	1.402 (6)	O1W—H1WB	0.8501
C25—H25A	0.9300	O4W—H4WA	0.8501
C13—C14	1.390 (6)	O4W—H4WB	0.8500
C13—H13A	0.9300	O5W—H5WA	0.8502
C24—C23	1.390 (6)	O5W—H5WB	0.8498
C24—H24A	0.9300	O6W—H6WA	0.8503
C32—C34	1.393 (6)	O6W—H6WB	0.8500
C32—C31	1.420 (6)	O7W—H7WA	0.8500
C1—C2	1.403 (5)	O7W—H7WB	0.8502
C1—H1A	0.9300	O8W—H8WA	0.8498
C11—C10	1.364 (7)	O8W—H8WB	0.8500
C11—C12	1.396 (6)	O9W—H9WA	0.8500
C11—H11A	0.9300	O9W—H9WB	0.8499
C28—C27	1.394 (6)	O10W—H10W	0.8500
C28—C30	1.418 (6)	O10W—H10E	0.8500
C12—H12A	0.9300	O2W—H2WA	0.8500
C14—C15	1.360 (6)	O2W—H2WB	0.8503
C14—H14A	0.9300		
			
N1—Zn1—N6	98.12 (11)	N1—C12—C11	123.3 (4)
N1—Zn1—N5	102.04 (11)	N1—C12—H12A	118.3
N6—Zn1—N5	77.29 (11)	C11—C12—H12A	118.3
N1—Zn1—N4	92.98 (11)	C15—C14—C13	119.8 (4)
N6—Zn1—N4	93.51 (11)	C15—C14—H14A	120.1
N5—Zn1—N4	163.28 (11)	C13—C14—H14A	120.1
N1—Zn1—N2	76.97 (11)	C2—C3—C4	119.8 (4)
N6—Zn1—N2	169.86 (10)	C2—C3—H3A	120.1
N5—Zn1—N2	94.92 (11)	C4—C3—H3A	120.1
N4—Zn1—N2	95.59 (10)	C42—C41—C40	121.2 (4)
N1—Zn1—N3	163.66 (11)	C42—C41—H41A	119.4
N6—Zn1—N3	95.37 (10)	C40—C41—H41A	119.4
N5—Zn1—N3	89.87 (11)	C30—C31—C32	122.1 (4)
N4—Zn1—N3	76.98 (11)	C30—C31—H31A	119.0
N2—Zn1—N3	91.04 (10)	C32—C31—H31A	119.0
C45—Se1—C43	85.43 (19)	C10—C9—C6	116.7 (4)
C12—N1—C6	117.7 (3)	C10—C9—C8	124.0 (4)
C12—N1—Zn1	128.0 (3)	C6—C9—C8	119.2 (4)
C6—N1—Zn1	114.1 (2)	C45—C44—N7	116.9 (4)
C13—N3—C17	118.3 (3)	C45—C44—C46	124.9 (4)
C13—N3—Zn1	128.1 (3)	N7—C44—C46	118.2 (4)
C17—N3—Zn1	113.5 (2)	C35—C34—C32	121.1 (4)
C24—N4—C18	118.4 (3)	C35—C34—H34A	119.5
C24—N4—Zn1	128.3 (2)	C32—C34—H34A	119.5
C18—N4—Zn1	113.2 (2)	C19—C20—C21	121.6 (4)
N7—C43—C40	124.8 (3)	C19—C20—H20A	119.2
N7—C43—Se1	113.4 (3)	C21—C20—H20A	119.2
C40—C43—Se1	121.8 (3)	C3—C2—C1	119.1 (4)
C1—N2—C5	118.0 (3)	C3—C2—H2A	120.4
C1—N2—Zn1	128.6 (2)	C1—C2—H2A	120.4
C5—N2—Zn1	113.3 (2)	C27—C26—C25	119.6 (4)
N4—C18—C21	121.9 (3)	C27—C26—H26A	120.2
N4—C18—C17	118.5 (3)	C25—C26—H26A	120.2
C21—C18—C17	119.7 (3)	C44—C45—Se1	110.7 (3)
C25—N5—C29	117.9 (3)	C44—C45—H45A	124.7
C25—N5—Zn1	128.4 (3)	Se1—C45—H45A	124.7
C29—N5—Zn1	112.9 (2)	O1—C46—O2	125.0 (5)
C36—N6—C33	117.9 (3)	O1—C46—C44	116.9 (5)
C36—N6—Zn1	128.7 (3)	O2—C46—C44	118.1 (4)
C33—N6—Zn1	113.1 (2)	C31—C30—C28	120.8 (4)
N5—C29—C28	123.0 (3)	C31—C30—H30A	119.6
N5—C29—C33	117.3 (3)	C28—C30—H30A	119.6
C28—C29—C33	119.7 (3)	C23—C22—C21	120.0 (4)
N3—C17—C16	122.5 (3)	C23—C22—H22A	120.0
N3—C17—C18	117.7 (3)	C21—C22—H22A	120.0
C16—C17—C18	119.7 (3)	C34—C35—C36	119.0 (4)
N2—C5—C4	122.6 (3)	C34—C35—H35A	120.5
N2—C5—C6	117.4 (3)	C36—C35—H35A	120.5
C4—C5—C6	120.0 (3)	C38—C39—C40	120.2 (4)
N6—C33—C32	123.0 (3)	C38—C39—H39A	119.9
N6—C33—C29	117.8 (3)	C40—C39—H39A	119.9
C32—C33—C29	119.2 (3)	C22—C23—C24	119.5 (4)
C5—C4—C3	117.3 (3)	C22—C23—H23A	120.3
C5—C4—C7	119.1 (4)	C24—C23—H23A	120.3
C3—C4—C7	123.6 (4)	C37—C42—C41	120.3 (4)
C43—N7—C44	113.6 (3)	C37—C42—H42A	119.8
C22—C21—C18	117.5 (3)	C41—C42—H42A	119.8
C22—C21—C20	123.9 (4)	C14—C15—C16	119.2 (4)
C18—C21—C20	118.6 (4)	C14—C15—H15A	120.4
C17—C16—C15	117.4 (4)	C16—C15—H15A	120.4
C17—C16—C19	119.6 (4)	C8—C7—C4	121.3 (4)
C15—C16—C19	123.0 (4)	C8—C7—H7A	119.4
C41—C40—C39	117.9 (4)	C4—C7—H7A	119.4
C41—C40—C43	122.3 (4)	C20—C19—C16	120.7 (4)
C39—C40—C43	119.8 (3)	C20—C19—H19A	119.7
N6—C36—C35	122.6 (4)	C16—C19—H19A	119.7
N6—C36—H36A	118.7	C11—C10—C9	119.8 (4)
C35—C36—H36A	118.7	C11—C10—H10A	120.1
N1—C6—C9	123.2 (3)	C9—C10—H10A	120.1
N1—C6—C5	117.8 (3)	C26—C27—C28	119.8 (4)
C9—C6—C5	119.0 (3)	C26—C27—H27A	120.1
N5—C25—C26	122.5 (4)	C28—C27—H27A	120.1
N5—C25—H25A	118.8	C7—C8—C9	121.4 (4)
C26—C25—H25A	118.8	C7—C8—H8A	119.3
N3—C13—C14	122.7 (4)	C9—C8—H8A	119.3
N3—C13—H13A	118.6	C42—C37—C38	119.7 (4)
C14—C13—H13A	118.6	C42—C37—H37A	120.2
N4—C24—C23	122.8 (4)	C38—C37—H37A	120.2
N4—C24—H24A	118.6	C39—C38—C37	120.6 (4)
C23—C24—H24A	118.6	C39—C38—H38A	119.7
C34—C32—C33	116.3 (4)	C37—C38—H38A	119.7
C34—C32—C31	124.8 (4)	H3WA—O3W—H3WB	125.7
C33—C32—C31	118.8 (4)	H1WA—O1W—H1WB	112.6
N2—C1—C2	123.1 (4)	H4WA—O4W—H4WB	103.4
N2—C1—H1A	118.4	H5WA—O5W—H5WB	114.4
C2—C1—H1A	118.4	H6WA—O6W—H6WB	118.3
C10—C11—C12	119.2 (4)	H7WA—O7W—H7WB	95.6
C10—C11—H11A	120.4	H8WA—O8W—H8WB	109.3
C12—C11—H11A	120.4	H9WA—O9W—H9WB	92.4
C27—C28—C29	117.1 (4)	H10W—O10W—H10E	88.7
C27—C28—C30	123.6 (4)	H2WA—O2W—H2WB	117.0
C29—C28—C30	119.3 (4)		
			
N6—Zn1—N1—C12	9.1 (3)	N4—C18—C21—C20	−178.2 (3)
N5—Zn1—N1—C12	87.8 (3)	C17—C18—C21—C20	2.5 (5)
N4—Zn1—N1—C12	−84.9 (3)	N3—C17—C16—C15	0.3 (5)
N2—Zn1—N1—C12	−179.9 (3)	C18—C17—C16—C15	−179.9 (3)
N3—Zn1—N1—C12	−136.2 (4)	N3—C17—C16—C19	−179.2 (3)
N6—Zn1—N1—C6	−176.4 (2)	C18—C17—C16—C19	0.5 (5)
N5—Zn1—N1—C6	−97.8 (2)	N7—C43—C40—C41	−172.0 (4)
N4—Zn1—N1—C6	89.6 (2)	Se1—C43—C40—C41	6.7 (5)
N2—Zn1—N1—C6	−5.4 (2)	N7—C43—C40—C39	8.1 (6)
N3—Zn1—N1—C6	38.2 (5)	Se1—C43—C40—C39	−173.2 (3)
N1—Zn1—N3—C13	−126.9 (4)	C33—N6—C36—C35	−0.3 (5)
N6—Zn1—N3—C13	87.5 (3)	Zn1—N6—C36—C35	−173.8 (3)
N5—Zn1—N3—C13	10.3 (3)	C12—N1—C6—C9	−0.2 (5)
N4—Zn1—N3—C13	179.9 (3)	Zn1—N1—C6—C9	−175.3 (3)
N2—Zn1—N3—C13	−84.6 (3)	C12—N1—C6—C5	−179.4 (3)
N1—Zn1—N3—C17	50.6 (5)	Zn1—N1—C6—C5	5.5 (4)
N6—Zn1—N3—C17	−95.0 (2)	N2—C5—C6—N1	−1.3 (5)
N5—Zn1—N3—C17	−172.2 (2)	C4—C5—C6—N1	178.2 (3)
N4—Zn1—N3—C17	−2.6 (2)	N2—C5—C6—C9	179.4 (3)
N2—Zn1—N3—C17	92.9 (2)	C4—C5—C6—C9	−1.1 (5)
N1—Zn1—N4—C24	16.8 (3)	C29—N5—C25—C26	−1.5 (6)
N6—Zn1—N4—C24	−81.5 (3)	Zn1—N5—C25—C26	167.0 (3)
N5—Zn1—N4—C24	−137.3 (4)	C17—N3—C13—C14	−1.1 (5)
N2—Zn1—N4—C24	94.0 (3)	Zn1—N3—C13—C14	176.2 (3)
N3—Zn1—N4—C24	−176.2 (3)	C18—N4—C24—C23	0.1 (5)
N1—Zn1—N4—C18	−165.4 (2)	Zn1—N4—C24—C23	177.9 (3)
N6—Zn1—N4—C18	96.3 (2)	N6—C33—C32—C34	1.4 (5)
N5—Zn1—N4—C18	40.5 (5)	C29—C33—C32—C34	−178.4 (3)
N2—Zn1—N4—C18	−88.2 (2)	N6—C33—C32—C31	−178.2 (3)
N3—Zn1—N4—C18	1.6 (2)	C29—C33—C32—C31	2.0 (5)
C45—Se1—C43—N7	1.8 (3)	C5—N2—C1—C2	0.2 (5)
C45—Se1—C43—C40	−177.1 (3)	Zn1—N2—C1—C2	−175.9 (3)
N1—Zn1—N2—C1	−179.0 (3)	N5—C29—C28—C27	0.8 (5)
N6—Zn1—N2—C1	−117.1 (6)	C33—C29—C28—C27	179.5 (3)
N5—Zn1—N2—C1	−77.7 (3)	N5—C29—C28—C30	−177.8 (3)
N4—Zn1—N2—C1	89.3 (3)	C33—C29—C28—C30	0.9 (5)
N3—Zn1—N2—C1	12.2 (3)	C6—N1—C12—C11	−1.0 (6)
N1—Zn1—N2—C5	4.8 (2)	Zn1—N1—C12—C11	173.4 (3)
N6—Zn1—N2—C5	66.7 (7)	C10—C11—C12—N1	1.3 (6)
N5—Zn1—N2—C5	106.0 (2)	N3—C13—C14—C15	0.3 (6)
N4—Zn1—N2—C5	−87.0 (2)	C5—C4—C3—C2	−0.9 (6)
N3—Zn1—N2—C5	−164.0 (2)	C7—C4—C3—C2	179.9 (4)
C24—N4—C18—C21	−1.6 (5)	C39—C40—C41—C42	−0.1 (6)
Zn1—N4—C18—C21	−179.7 (2)	C43—C40—C41—C42	−180.0 (4)
C24—N4—C18—C17	177.7 (3)	C34—C32—C31—C30	−179.5 (4)
Zn1—N4—C18—C17	−0.4 (3)	C33—C32—C31—C30	0.1 (6)
N1—Zn1—N5—C25	83.7 (3)	N1—C6—C9—C10	0.9 (5)
N6—Zn1—N5—C25	179.5 (3)	C5—C6—C9—C10	−179.9 (3)
N4—Zn1—N5—C25	−122.7 (4)	N1—C6—C9—C8	−177.6 (3)
N2—Zn1—N5—C25	6.0 (3)	C5—C6—C9—C8	1.6 (5)
N3—Zn1—N5—C25	−85.0 (3)	C43—N7—C44—C45	2.2 (5)
N1—Zn1—N5—C29	−107.3 (2)	C43—N7—C44—C46	−179.1 (3)
N6—Zn1—N5—C29	−11.6 (2)	C33—C32—C34—C35	−0.2 (6)
N4—Zn1—N5—C29	46.2 (5)	C31—C32—C34—C35	179.5 (4)
N2—Zn1—N5—C29	175.0 (2)	C22—C21—C20—C19	178.7 (4)
N3—Zn1—N5—C29	84.0 (2)	C18—C21—C20—C19	−0.7 (5)
N1—Zn1—N6—C36	−75.9 (3)	C4—C3—C2—C1	1.4 (6)
N5—Zn1—N6—C36	−176.5 (3)	N2—C1—C2—C3	−1.1 (6)
N4—Zn1—N6—C36	17.6 (3)	N5—C25—C26—C27	0.4 (7)
N2—Zn1—N6—C36	−136.1 (6)	N7—C44—C45—Se1	−0.7 (5)
N3—Zn1—N6—C36	94.9 (3)	C46—C44—C45—Se1	−179.3 (3)
N1—Zn1—N6—C33	110.4 (2)	C43—Se1—C45—C44	−0.5 (3)
N5—Zn1—N6—C33	9.8 (2)	C45—C44—C46—O1	159.8 (5)
N4—Zn1—N6—C33	−156.1 (2)	N7—C44—C46—O1	−18.7 (6)
N2—Zn1—N6—C33	50.1 (7)	C45—C44—C46—O2	−19.6 (7)
N3—Zn1—N6—C33	−78.9 (2)	N7—C44—C46—O2	161.9 (4)
C25—N5—C29—C28	0.8 (5)	C32—C31—C30—C28	−1.7 (6)
Zn1—N5—C29—C28	−169.4 (3)	C27—C28—C30—C31	−177.3 (4)
C25—N5—C29—C33	−177.9 (3)	C29—C28—C30—C31	1.2 (6)
Zn1—N5—C29—C33	11.9 (4)	C18—C21—C22—C23	−1.6 (5)
C13—N3—C17—C16	0.8 (5)	C20—C21—C22—C23	179.0 (4)
Zn1—N3—C17—C16	−176.9 (2)	C32—C34—C35—C36	−1.2 (6)
C13—N3—C17—C18	−179.0 (3)	N6—C36—C35—C34	1.5 (6)
Zn1—N3—C17—C18	3.3 (3)	C41—C40—C39—C38	0.3 (6)
N4—C18—C17—N3	−2.0 (4)	C43—C40—C39—C38	−179.8 (4)
C21—C18—C17—N3	177.3 (3)	C21—C22—C23—C24	0.2 (6)
N4—C18—C17—C16	178.2 (3)	N4—C24—C23—C22	0.6 (6)
C21—C18—C17—C16	−2.4 (5)	C40—C41—C42—C37	−0.6 (6)
C1—N2—C5—C4	0.3 (5)	C13—C14—C15—C16	0.9 (6)
Zn1—N2—C5—C4	177.0 (3)	C17—C16—C15—C14	−1.2 (5)
C1—N2—C5—C6	179.8 (3)	C19—C16—C15—C14	178.4 (4)
Zn1—N2—C5—C6	−3.5 (4)	C5—C4—C7—C8	0.9 (6)
C36—N6—C33—C32	−1.1 (5)	C3—C4—C7—C8	−179.9 (4)
Zn1—N6—C33—C32	173.3 (3)	C21—C20—C19—C16	−1.3 (6)
C36—N6—C33—C29	178.6 (3)	C17—C16—C19—C20	1.4 (5)
Zn1—N6—C33—C29	−6.9 (4)	C15—C16—C19—C20	−178.2 (4)
N5—C29—C33—N6	−3.4 (4)	C12—C11—C10—C9	−0.5 (6)
C28—C29—C33—N6	177.8 (3)	C6—C9—C10—C11	−0.5 (6)
N5—C29—C33—C32	176.3 (3)	C8—C9—C10—C11	178.0 (4)
C28—C29—C33—C32	−2.4 (5)	C25—C26—C27—C28	1.3 (7)
N2—C5—C4—C3	0.0 (5)	C29—C28—C27—C26	−1.8 (6)
C6—C5—C4—C3	−179.4 (3)	C30—C28—C27—C26	176.8 (4)
N2—C5—C4—C7	179.3 (3)	C4—C7—C8—C9	−0.4 (7)
C6—C5—C4—C7	−0.2 (5)	C10—C9—C8—C7	−179.3 (4)
C40—C43—N7—C44	176.2 (3)	C6—C9—C8—C7	−0.9 (6)
Se1—C43—N7—C44	−2.6 (4)	C41—C42—C37—C38	1.1 (7)
N4—C18—C21—C22	2.4 (5)	C40—C39—C38—C37	0.2 (7)
C17—C18—C21—C22	−176.9 (3)	C42—C37—C38—C39	−0.9 (7)

### Hydrogen-bond geometry (Å, °)

**Table:**

*D*—H···*A*	*D*—H	H···*A*	*D*···*A*	*D*—H···*A*
O1W—H1WA···O2W^i^	0.85	2.05	2.898 (5)	179
O1W—H1WB···O9W^ii^	0.85	1.98	2.830 (6)	180
O4W—H4WA···N7^ii^	0.85	2.22	3.044 (5)	163
O4W—H4WA···O1^ii^	0.85	2.40	2.921 (7)	120
O5W—H5WA···O6W^iii^	0.85	1.91	2.762 (6)	178
O6W—H6WA···Cl1^iii^	0.85	2.24	3.076 (5)	168
O7W—H7WB···Cl1^iv^	0.85	2.54	3.391 (5)	179.
O8W—H8WB···O7W^v^	0.85	1.97	2.817 (7)	180
O2W—H2WA···O9W^vi^	0.85	1.96	2.806 (5)	179
O2W—H2WB···Cl1^vii^	0.85	2.28	3.129 (3)	180
O4W—H4WB···O1	0.85	1.98	2.809 (6)	166
O5W—H5WB···O3W	0.85	2.07	2.915 (6)	173
O6W—H6WB···O2	0.85	2.05	2.892 (6)	171
O7W—H7WA···Cl1	0.85	2.24	3.006 (5)	150
O8W—H8WA···O2	0.85	1.97	2.819 (5)	172
O9W—H9WA···O8W	0.85	2.05	2.864 (8)	161
O9W—H9WB···O10W	0.85	2.18	3.030 (8)	178
O10W—H10W···O4W	0.85	2.02	2.866 (8)	173
O10W—H10E···O2	0.85	2.08	2.925 (6)	171

Symmetry codes: (i) *x*+1, *y*, *z*; (ii) −*x*+2, −*y*, −*z*+1; (iii) −*x*+1, −*y*+1, −*z*+1; (iv) −*x*+1, −*y*+1, −*z*+2; (v) *x*, *y*, *z*−1; (vi) *x*−1, *y*, *z*+1; (vii) *x*−1, *y*, *z*.
